# The inflammatory microenvironment in vestibular schwannoma

**DOI:** 10.1093/noajnl/vdaa023

**Published:** 2020-02-27

**Authors:** Cathal John Hannan, Daniel Lewis, Claire O’Leary, Carmine A Donofrio, Dafydd Gareth Evans, Federico Roncaroli, David Brough, Andrew Thomas King, David Coope, Omar Nathan Pathmanaban

**Affiliations:** 1 Manchester Centre for Clinical Neurosciences, Salford Royal Foundation Trust, Manchester Academic Health Science Centre, Manchester, UK; 2 Division of Neuroscience & Experimental Psychology, School of Biological Sciences, Faculty of Biology Medicine and Health, University of Manchester, Manchester, UK; 3 Manchester Centre for Genomic Medicine, St Mary’s Hospital, Manchester University Hospitals National Health Service Foundation Trust, Manchester, UK; 4 Division of Evolution & Genomic Sciences, School of Biological Sciences, Faculty of Biology Medicine and Health, University of Manchester, Manchester, UK; 5 Division of Cardiovascular Sciences, School of Medical Sciences, Faculty of Biology Medicine and Health, University of Manchester, Manchester, UK; 6 Division of Cell Matrix Biology & Regenerative Medicine, School of Biological Sciences, Faculty of Biology Medicine and Health, University of Manchester, Manchester, UK

**Keywords:** biomarkers, inflammation, macrophages, neurofibromatosis type 2, vestibular schwannoma

## Abstract

Vestibular schwannomas are tumors arising from the vestibulocochlear nerve at the cerebellopontine angle. Their proximity to eloquent brainstem structures means that the pathology itself and the treatment thereof can be associated with significant morbidity. The vast majority of these tumors are sporadic, with the remainder arising as a result of the genetic syndrome Neurofibromatosis Type 2 or, more rarely, *LZTR1-*related schwannomatosis. The natural history of these tumors is extremely variable, with some tumors not displaying any evidence of growth, others demonstrating early, persistent growth and a small number growing following an extended period of indolence. Emerging evidence now suggests that far from representing Schwann cell proliferation only, the tumor microenvironment is complex, with inflammation proposed to play a key role in their growth. In this review, we provide an overview of this new evidence, including the role played by immune cell infiltration, the underlying molecular pathways involved, and biomarkers for detecting this inflammation in vivo. Given the limitations of current treatments, there is a pressing need for novel therapies to aid in the management of this condition, and we conclude by proposing areas for future research that could lead to the development of therapies targeted toward inflammation in vestibular schwannoma.

Vestibular schwannomas (VS) are histologically benign tumors, which arise from the myelin-forming Schwann cells lining the vestibulocochlear or eighth cranial nerve. They account for approximately 8% of all intracranial tumors and with the advent and widespread adoption of magnetic resonance imaging (MRI), their incidence has risen significantly to around 15–20 per million per year.^1,2^ While 95% of these tumors occur as a unilateral, sporadic entity they can also occur bilaterally as part of the dominantly inherited tumor syndrome neurofibromatosis type 2 (NF2) or, more rarely, unilaterally due to *LZTR1-*related schwannomatosis.^1,3^ Presently, there are 3 main management strategies for sporadic VS: radiological observation, stereotactic radiosurgery, and microsurgical resection. Management of these tumors can be problematic, however, due to their unpredictable and sometimes rapid growth, and the threat to other cranial nerves, notably the facial nerve. In NF2, the involvement of both vestibulocochlear nerves by bilateral VS makes their treatment especially challenging.^4–6^ There is therefore a pressing need for novel therapeutics in the management of these tumors, but with the exception of bevacizumab in NF2, no therapies specifically targeted to the aberrant processes that underlie the development and progression of VS exist.^7^ Mutations of the *Nf2* gene on chromosome 22 and subsequent loss of the tumour-suppresor protein Merlin are thought to be the primary molecular events responsible for inducing Schwann cell neoplasia in both sporadic and NF2 related VS.^8,9^ While considerable progress has been made in deciphering the downstream molecular pathways involved,^9,10^ outstanding questions remain such as how these molecular alterations at the Schwann cell level translate into schwannoma formation in vivo and the highly varied growth rates seen within these tumors.^11^ A greater understanding of the tumor microenvironment in VS is required and there is now growing evidence from ex vivo and in vivo studies that one aspect of this microenvironment, inflammation, may be critically important in driving tumor behavior. The purpose of this review is to therefore summarize recent evidence on the inflammatory microenvironment within VS, with a view to firstly identifying deficiencies in our current understanding of the pathophysiological processes involved and secondly establish areas for further research that may lead to immune targeting therapies against these tumors.

## Immune Cell Infiltration in VS

It has long been recognized that immune cell infiltrates in VS, including macrophages, B and T lymphocytes are widespread, especially within the loosely cellular, so-called Antoni type B areas.^12–16^ In an earlier study of this inflammatory microenvironment, Labit-Bouvier et al.^17^ measured the extent of immune cell infiltrates in 69 sporadic VS tissue specimens and demonstrated that the extent of intratumoral immune cell infiltrates, as measured through immunostaining for the common leukocyte antigen CD45, correlated with the duration of clinical symptoms. To date, the exact role that this immune cell population plays in VS pathogenesis and progression is unknown and one component of the immune cell infiltrate in VS, which has received increased attention, is the role of tumor-associated macrophages (TAMs).^18–20^

TAMs are demonstrated to be key drivers in the growth and progression of many solid organ tumors.^21,22^ They are thought to predominantly arise from circulating bone marrow-derived monocytes and within the tumor microenvironment, they regulate many pathophysiological processes including tumor growth, tumor invasion, and angiogenesis.^21,22^ TAMs display a wide range of functions depending on the specific site upon which they act and rather than forming a monolithic population of cells they display marked heterogeneity in both their expression profiles and activity.^22,23^ Differential cytokine expression within the tumor microenvironment leads to this spectrum of functional macrophage states, but at the extremes of this spectrum TAMs can be broadly divided into 2 groups, the pro-inflammatory classically activated M1-type macrophages and the immune regulating, alternately activated M2-type macrophages.^23^ M1-like macrophages are activated by interferon-γ and lipopolysaccharide and secrete pro-inflammatory cytokines including IL-1 and IL-6. They have demonstrable tumoricidal activity in certain types of cancer and indeed their presence in certain non-CNS tumor types has been associated with a better prognosis.^24,25^ M2-like macrophages by contrast are induced by secreted IL-10, IL-13, and glucocorticoid hormones and have been shown to be pro-tumorigenic in vivo, both attenuating tumoricidal activity and facilitating angiogenesis.^26^ M1- and M2-type macrophages can be partly differentiated based upon their cell surface marker expression.^23^ One purported cell surface marker for M2 macrophages is CD163,^27^ the scavenger receptor for the hemoglobin–haptoglobin complex, and studies in non-CNS tumors such as gastric, colorectal, and pancreatic cancers have demonstrated that infiltration of CD163+ M2-type macrophages is associated with tumor progression and overall poorer prognosis.^28–30^

## The Role of TAMs in VS Growth

**Fig. 1:**
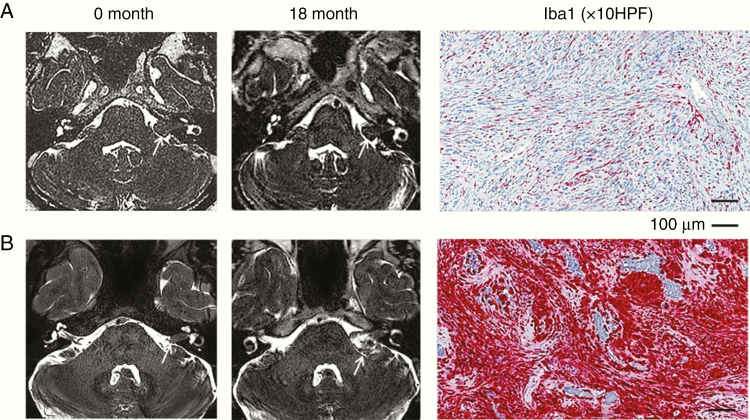
Iba1+ macrophage abundance in growing vestibular schwannoma (VS). Representative serial MRI and Iba1-stained immunosections from a patient with a static VS (A) and a rapidly growing sporadic VS (B). Serial T2W imaging through the cerebellopontine angle at 0 months and 18 months demonstrates the differential growth rates between the two tumors and Iba1 immunostains (red, immunoperoxidase, ×10) demonstrate the high macrophagic infiltrates seen within the rapidly growing VS.

Recent ex vivo and in vivo evidence has suggested that TAMs may play a key role in the progression and growth of VS.^18,19,31^ In 2 histological studies, de Vries et al.^18,31^ examined the expression of the macrophage markers CD68 and CD163 in VS tissue and demonstrated not only a positive relationship with tumor growth but also a positive correlation between tissue microvessel density and CD163+ expression, in keeping with the capacity of CD163+ M2 macrophages to induce angiogenesis. A more recent immunohistochemical analysis of VS tissue, obtained from patients undergoing a subtotal resection of their tumor, also demonstrated higher macrophage infiltration in tumors that went on to demonstrate postoperative progression.^32^ Interestingly, however, the authors reported a negative association between M2 macrophage infiltration and the risk of postoperative progression.^32^ While the number of samples in the aforementioned studies are small and their outcome measures (preoperative tumor growth rate and the risk of postoperative progression) differ, the apparent contradiction in their findings nonetheless highlights the evolving nature of our understanding of the role of macrophages in VS pathophysiology and perhaps the oversimplification that is the M1/M2 dichotomy.^33^ In alignment with the studies above, Lewis et al.^19^ in a combined imaging and neuropathology study demonstrated greater Iba1+ macrophage infiltration in growing VS compared to static tumors (Figure 1) and showed that the majority of proliferating cells (50–70%) within growing tumors were in fact not Schwann cells, but Iba1+ macrophages, giving further credence to the argument that macrophage infiltration plays a significant role in sporadic VS growth.

There has been comparatively less research into the role of TAMs in NF2-related VS, but small histopathological studies^34,35^ have demonstrated Iba1 and CD68-positive macrophage occurrence among human schwannoma samples. In one such study, Wang et al.^35^ characterized the tumor immune microenvironment in 10 NF2-related schwannomas by immunohistochemistry and demonstrated not only the expression of the macrophage marker CD68 but also the expression of the T lymphocyte markers CD3 and CD8. The authors noted that while CD20+ B lymphocytes were also present in the tumor microenvironment, they were much sparser in number and predominantly perivascular in location. A summary of ex vivo studies examining macrophage and immune cell expression in VS is provided in Table 1. While it is apparent that TAMs are a pathological feature in VS, particularly in tumors with high growth rates, the exact role that they play in tumor progression remains unclear. While it is possible that this macrophage infiltration occurs as a result of an inflammatory response to tumor growth or angiogenesis, it seems likely that macrophage burden is at least contributory to the regulation of the factors that drive tumor growth. Further mechanistic studies directed at elucidating the exact role that TAMs play in VS are required.

**Table 1:** Recent Ex Vivo Tissue Studies Examining Immune Cell Populations in vestibular schwannoma (VS)

Author	Year	*N*	Inflammatory Cell Marker Studied	Key Findings
Labit- Bouvier et al.	2000	69 sporadic VS	CD45	• CD45 index significantly correlated with both morphological evaluation of inflammation and duration of symptoms when lasting >1 year • Clinical growth index was significantly positively correlated with microvessel density
de Vries et al.	2012	67 sporadic VS	CD45, CD68	• CD45 and CD68 expression correlated with tumor size, tumor growth index, and CD31+ microvessel density • Tumors with a high number of CD68+ cells displayed a significantly higher microvascular density than tumors with low/absent CD68+ cells
de Vries et al.	2013	10 fast-growing and 10 slow- growing sporadic VS	CD163	• CD163 expression and microvessel density were significantly higher in a cohort of 10 fast-growing VS compared to a slow-growing VS cohort • Tumors with higher CD163 expression had significantly greater microvessel density
Schulz et al.	2016	30 sporadic, 10 NF2-related VS	CD68, Iba1 MMR (macrophage mannose receptor, CD206)	• CD68 and Iba1 expression was found in 28/30 sporadic VS, 9/10 NF2-related schwannomas, and 4/4 Schwann cell tumors associated with schwannomatosis • M2-type macrophage marker MMR/CD206 was expressed in both sporadic and NF2-associated schwannomas
Lewis et al.	2018	8 sporadic VS	Iba1	• Greater Iba1+ macrophage infiltration in growing VS compared to static tumors • Within growing VS, Iba1+ macrophages accounted for the majority (50–70%) of cells • Growing tumors demonstrated a significantly higher percentage of inflammatory Ki-67+/Iba1+ cells • Iba1+ macrophages accounted for >50% of the Ki67+ cells within these lesions
Wang et al.	2018	10 NF2-related VS	CD3, CD20, CD8, and CD68	• Sparse to moderate presence of CD68, CD3, and CD8 in 9/10 NF2-related VS studied • CD20+ B lymphocytes were either absent (9/10 samples) or sparsely present in perivascular regions (1/10)
Sagers et al.	2019	22 sporadic VS, 7 control great auricular nerves (GANs)	CD68	• 19/ 22 VS demonstrated moderate to high immunohistochemical staining for CD68+ macrophages • Using blinded semiquantitative scoring, VS from patients with poor hearing demonstrated a nonsignificant trend toward increased CD68 positivity compared to patients with good hearing
Perry et al.	2019	44 sporadic VS undergoing subtotal resection (STR)	CD68, CD163, and PD-L1	• Significantly increased CD68 macrophage density among tumors that progressed and patients who had an unfavorable House–Brackmann grade III–VI facial nerve post STR • Compared to tumors that progressed, CD163 percent positivity and M2 index (no. of CD163+ cells/no. of CD68+ cells) were significantly increased among tumors that remained stable post STR • PD-L1 percent positivity was significantly elevated in both tumors that progressed and tumors associated with an unfavorable facial nerve outcome (HB III–VI) post STR

## Cytokine/Chemokine Expression Profiles in VS

**Fig. 2:**
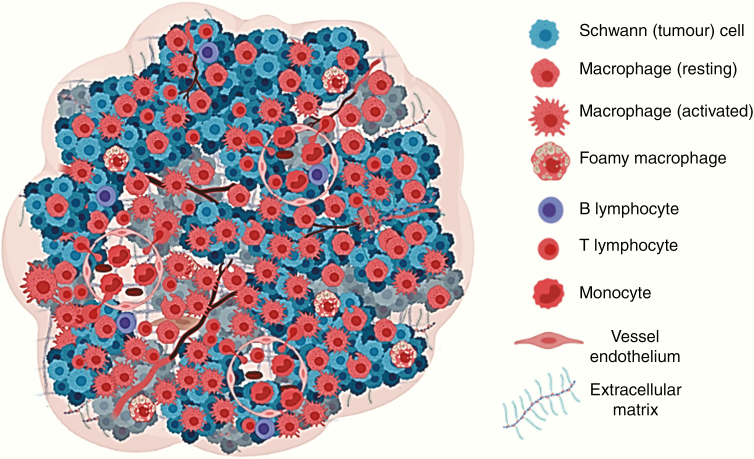
Immune microenvironment in vestibular schwannoma (VS). Alongside the neoplastic Schwann cell population studies have shown that immune cells also contribute to the tumor microenvironment (TME) in VS. This immune cell population includes both B and T lymphocytes and a population of tumor-associated macrophages (TAMs).^12–16^ Circulating bone marrow-derived monocytes are thought to be the origin of these TAMs and previous studies have demonstrated a close association between the tumor vasculature and this intratumoral macrophage population in VS.^18,19,31^ While recent studies have demonstrated upregulation of candidate cytokines, chemokines, and cellular adhesion molecules within sporadic VS tissue,^38,44,46,47^ the key chemoattractants that recruit circulating monocytes and drive their differentiation into macrophages are still under investigation. Extracellular matrix (ECM) components such as collagen and glycoprotein laminin are also a prominent feature of the TME in VS.^12,13^ The extent to which the ECM interacts with the immune cell population in driving VS progression has not been investigated, however, and should be a direction for future research. This figure was created under license using BioRender.com. https://biorender.com/

Data from ex vivo tissue specimens therefore suggest that immune cell infiltration is a key component of the tumor microenvironment in VS (Figure 2). To date, however, the key molecular pathways driving this inflammatory microenvironment have not been established. Perhaps the earliest demonstration that there is expression of immunogenic mediators within VS came from early in vitro leukocyte migration assays, which demonstrated that tumor extracts, serum, and cerebrospinal fluid from patients with VS could induce a cell-mediated immune response.^36,37^ More recently Taurone et al.^38^ in a small immunohistochemical study demonstrated that compared with normal vestibular nerve there is upregulation of the pro-inflammatory cytokines IL-1β, IL-6, and TNF-α within sporadic VS tissue. Upregulation of the leukocyte adhesion molecules ICAM-1 was also demonstrated in keeping with previous in vitro studies that have demonstrated TNF-α and IL-1β induced ICAM-1 expression on human Schwann cell lines.^39^ In line with these findings, Dilwali et al.^40^ demonstrated that secretions from resected sporadic VS tissue contained TNF-α, with higher levels of TNF-α secretion correlating with both poorer hearing among patient subjects and greater secretion-induced cellular loss in murine cochlear explants.

Within the tumor inflammatory microenvironment, the cytokines macrophage colony-stimulating factor (M-CSF) and IL-34 are thought to be important factors capable of polarizing macrophages toward a pro-tumorigenic M2-like phenotype^41,42^ and high expression levels of these cytokines are associated with disease progression and unfavorable outcome in several types of tumors.^42,43^ de Vries et al.^44^ semi-quantitatively examined the expression of M-CSF and IL-34 in resected VS tissue and while there was no relationship between IL-34 levels and tumor growth, fast-growing and cystic tumors did demonstrate greater expression levels of M-CSF, in line with their previous findings of increased CD163 macrophage abundance within this tumor cohort. Chemokines are a subgroup of cytokines that regulate chemotaxis of immune cells into the tumor microenvironment and they are also relevant in VS pathogenesis. Macrophage inflammatory protein 1α (MIP-1α) is a member of the C-C family of chemokines that regulates monocyte/macrophage chemotaxis and in an ex vivo tissue study of peripheral nerve schwannomas by Mori et al.,^45^ there was co-localization between MIP-1α and the macrophage marker CD68 in Antoni type B areas of myxoid degeneration. C-X-C family chemokines are also thought to be of relevance and previous studies demonstrated that compared to healthy nerves there is greater expression levels of chemokines such as CXCL16 and CXCL12 and their respective receptors within VS tissue.^46,47^ A summary of studies examining candidate cytokine/chemokine expression profiles in VS is provided in Table 2.

**Table 2:** Studies Examining Cytokine/Chemokine Profiles in VS

Author	Year	Study Design		Cytokines/Chemokines Studied	Key Findings
Held-Feindt et al.	2008	Ex vivo tissue study	21 sporadic VS, 9 normal control nerves	CXCL16, CXCR6/Bonzo (CXCL16 receptor)	• CXCL16 and CXCR6 mRNA expression levels were significantly elevated in VS samples compared to normal control nerves • CXCL16 protein abundance quantified using ELISA and Western blot techniques was significantly higher in VS compared to normal nerve tissues • CXCL16 and CXCR6 co-stained with S-100 Schwann cells on immunofluorescence but not with CD68 macrophages
Taurone et al.	2015	Ex vivo tissue study	10 sporadic VS, 10 normal vestibular nerves	IL-1β, IL-6, TNF-α, TGFβ ICAM-1	• TGF-β1, IL-1β, IL-6, TNF-α, and ICAM-1 exhibited increased expression in human VS tissue compared with normal vestibular nerve samples
Dilwali et al.	2015	Ex vivo study of VS secretions	13 sporadic VS	TNF-α	• Secretions from 9 tumors studied contained varying concentrations of TNF-α • Increased TNF-α levels in the secretions correlated with increased sensorineural hearing loss in the affected ear • Application of TNF-α to murine cochlear explants resulted in neurite loss and TNF neutralization partly prevented this loss
de Vries et al.	2018	Ex vivo tissue study	10 fast-growing, 10 slow-growing VS	M-CSF, IL-34	• All VS studied expressed IL-34 and M-CSF • Fast-growing and cystic tumors demonstrated greater expression levels of M-CSF compared to slow-growing and non-cystic tumors, respectively • CD163 expression was higher in tumors with strong M-CSF expression
Breun et al.	2018	Ex vivo tissue study	30 sporadic VS, 30 NF2-related VS, 10 control nerve samples (4 sural nerve, 6 vestibular nerve)	CXCL12, CXCR4 (receptor)	• Sporadic VS samples demonstrated a 4.25-fold higher CXCR4 mRNA expression than control samples and in NF2-associated VS expression was 4.9-times higher compared to the control group • A nonsignificant trend toward higher CXCR4 expression levels in patients with greater hearing impairment. Tumor growth patterns prior to surgery and tumor extension at the time of surgery did not correlate with the CXCR4 mRNA expression level. • Double immunofluorescence demonstrated that both CXCR4 and CXCL12, the CXCR4 ligand, were expressed mainly in S100 Schwann cells
Sagers et al.	2019	Ex vivo tissue study	30 sporadic VS samples (22 samples for IHC), 7 control great auricular nerves (GANs)	IL-1β, NLRP3 inflammasome	• VS from patients with poor hearing (*n* = 11) demonstrated a nonsignificant trend toward increased IL-1β and NLRP3 immunohistochemical staining compared to patients with good hearing (*n* = 11) on blinded semiquantitative scoring • Increased NLRP3, IL-18, and IL-1β RNA expression in patients with poor hearing (*n* = 15) compared to patients with good hearing (*n* = 15) and control nerves

A clear limitation in the current literature is that the exact cellular source of cytokines has not been established. While some tissue studies have demonstrated spatial co-localization between cytokines and S100+ Schwann cells,^46,47^ it is equally likely that many of the observed mediators also originate from the immune cell population itself.^48–50^ Previous authors have suggested that one of the primary events driving the immune microenvironment in VS may be cytokine upregulation following Schwann cell injury.^20,38,50^ Injury to peripheral nerves results in the infiltration of immune cells, including macrophages, and there is growing evidence that Schwann cells play an active role in driving this inflammation.^50^ In murine models of peripheral nerve injury, Schwann cells lining the sciatic nerve upregulate TNF-α expression following crush injury^49^ and primary denervated Schwann cells in culture induce macrophage chemotaxis through secretion of IL-6 and leukaemia inhibitory factor.^50^ Both loss of axonal contact and diffusible molecules released by degenerating nerve axons may regulate Schwann cell gene expression^50,51^ but the extent to which these injury repair mechanisms also operate in vestibular schwannoma is currently unclear. In an in vivo *Nf2* knockout model of schwannoma formation, mice bred to have a combined heterozygous *Nf2* deletion in both Schwann cells and neurons (P0-Cre;Nefh-Cre;Nf2fl/+) almost uniformly developed Sciatic nerve schwannomas following crush injury at 8 months of age. Within these schwannomas, the authors demonstrated not only prominent infiltration of arginase-1 expressing M2-type macrophages but also increased expression of numerous cytokines including IL-1, IL-6, and TNF-α within tumor lysates.^20^ The above-cited studies, however, focus on schwannoma formation in peripheral nerves and as such the applicability of these injury models to VS formation is currently unclear. Schwann cell *Nf2* conditional knockout and other genetically engineered mouse models of NF2 pathogenesis demonstrate that nerve injury is not an absolute requirement for cranial schwannoma formation in vivo^52^ and given their anatomical location, nonsurgical trauma to the vestibular nerves is extremely unlikely to occur. What these studies do suggest however is that Schwannoma formation can be induced following a noxious, pro-inflammatory stimulus. Further research is therefore required to better clarify the role of nerve injury and identify the key tumorigenic stimuli in early VS formation.

## Molecular Regulators of Immune Activation in VS

The development of novel immunomodulatory therapeutic approaches against VS first requires the characterization of the key molecular networks driving and regulating the tumor inflammatory microenvironment.

One such purported network is the nuclear factor kappa-B (NF-κB) pathway. NF-κB is a transcription factor that modulates a number of intracellular processes, including the regulation of cellular apoptosis and the transcriptional coordination of numerous immune-related genes including those encoding cytokines, chemokines, and cellular adhesion molecules.^53^ In vivo models of colonic and hepatocellular carcinoma have suggested that NF-κB signaling plays a key role in the maintenance of cancer-promoting inflammation,^54,55^ and in murine ovarian cancer models the polarization of TAMs to a pro-tumorigenic M2 phenotype is critically dependent on the IκB kinase-mediated activation of NF-κB.^56^ While it is likely that there are substantive differences in the microenvironment of benign and malignant tumors, there is evidence that the NF-κB pathway may also play a key role in driving benign tumor progression. Indeed in a murine model of cutaneous neurofibroma, a peripheral nerve sheath tumor distinct from Schwannoma, the p65 subunit of NF-κB was shown to promote immune cell-mediated tumor growth.^57^ The *Nf2* gene product Merlin has demonstrable activity as an inhibitor of NF-κB in murine fibroblast and rat glioma cells,^58^ and in human schwannoma cell lines Merlin has been shown to be a key negative regulator of NF-κB activity.^59^ In a network analysis of differentially expressed genes in VS, Dilwali et al.^60^ identified NF-κB as the “hub” of a significantly upregulated gene network and demonstrated that compared to normal human Schwann cells there was increased expression and transcription of genes encoding for subunits of the NF-κB transcription factor in both VS tissue and human VS-derived cell lines.

**Fig. 3:**
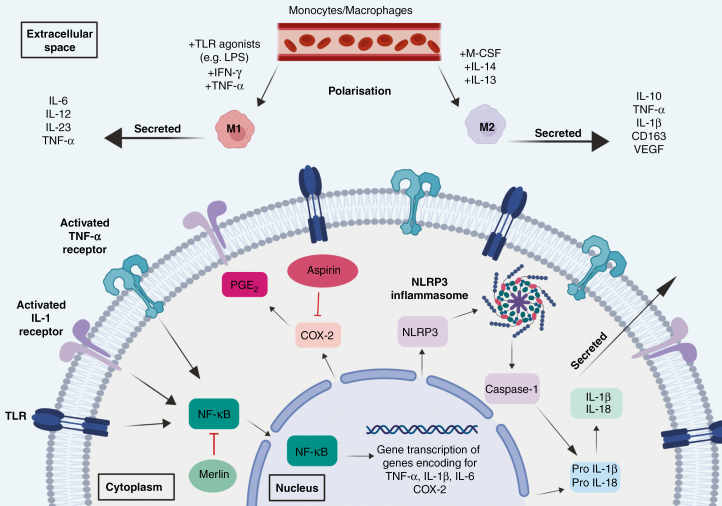
Hypothesized inflammatory pathways implicated in vestibular schwannoma. Typically macrophages are polarized to an M1 phenotype by TLR (Toll-like receptor) ligands such as LPS (lipopolysaccharide), IFNγ (interferon-γ), or TNFα (tumor necrosis factor-α), secrete pro-inflammatory cytokines, and have anti-tumorigenic properties. Alternatively, these macrophages can be polarized to an M2 phenotype by GM-CSF (granulocyte-macrophage colony-stimulating factor), IL-12 (interleukin-12), and IL-14 (interleukin-14), secrete anti-inflammatory cytokines, and have pro-tumorigenic properties. These secreted cytokines bind to receptors on the cell surface of Schwann cells, initiate the transcription factor NF-κB (nuclear factor kappa-B) upregulating TNF-α, IL-1β (interleukin-1β), IL-6 (interleukin-6), COX-2 (cyclooxygenase-2), NLRP3 (NOD-like receptor protein 3), pro-IL1β (pro-interleukin1β), and IL-18 (interleukin-18). The cytoskeleton protein Merlin can act as a tumor suppressor by blocking the transcription of nuclear NF-κB. COX-2 production can be regulated by NF-κB and inhibitors of COX-2 such as aspirin reduce its activation. This figure was created under license using BioRender.com. https://biorender.com/

NF-κB transcriptional activity is stimulated by various cytokines including TNF-α, IL-1, and IL-6,^58,61^ and there is evidence of a positive feedback, autocrine signaling loop in the regulation of NF-κB activity: NF-κB translocation to the nucleus leads to increased expression of genes encoding for TNF-α and IL-1, which in turn can stimulate increased NF-κB activity^62,63^ (Figure 3). Under physiological conditions, this positive feedback loop is balanced by equivalent negative regulators, but in systemic malignancies such as pancreatic and breast cancers, constitutively active NF-κB autocrine pathways have been demonstrated.^64,65^ Within VS, the role that these autocrine positive feedback loops play in cytokine generation, however, is yet to be explored. Studies of TAM behavior in vitro have demonstrated that NF-κB activation is a critical step in the M-CSF-driven polarization of M1 macrophages toward a pro-tumorigenic M2 phenotype,^66^ an interesting finding considering previous studies that demonstrated M-CSF expression and CD163+ M2-type macrophages within VS tissue.^18^ In the study by Dilwali et al.,^60^ use of specific NF-κB inhibitors and siRNA (short interfering RNA) against NF-κB constituent proteins selectively reduced the proliferation of VS cells in culture.^60^ The above studies also suggest that in addition to its antiproliferative effects, targeting NF-κB may additionally help downregulate pro-inflammatory cytokine pathways within the VS microenvironment and potentially induce macrophage polarization toward an antitumorigenic M1-type phenotype.^21^

Alongside the NF-κB pathway, the NLRP3 inflammasome has also recently emerged as a potentially important regulator of immune activation in VS. Inflammasomes are multi-protein complexes that act as important upstream regulators of IL-1β and IL-18 activation in immune cells^67^ and are activated by cytosolic pattern-recognition receptors.^68^ In a recent study comparing candidate gene expression in VS with that of normal peripheral nerve, the authors demonstrated upregulation of genes associated with the NLRP3 inflammasome in VS tissue. Increased expression of these genes in VS tissue derived from patients with poor hearing as against those with good hearing was also evident. Immunohistochemical analysis confirmed the presence of NLRP3-associated proteins, as well as macrophage infiltration, in the VS tissue.^69^ An overview of potential candidate inflammatory molecular pathways in VS is shown in Figure 3.

## Cyclooxygenase 2 Pathway in VS

One immune-related pathway that has received considerable attention as a potential therapeutic target in VS is the enzyme cyclooxygenase 2 (COX-2). This enzyme metabolizes arachidonic acid to prostaglandins, prostacyclin, and thromboxane and in contrast to its constitutively expressed counterpart isoenzyme, COX-1, it is an inducible enzyme found at sites of inflammation and neoplasia.^70^ COX-2 is overexpressed in a number of benign and malignant tumors, including those of the central nervous system, and higher levels of COX-2 expression have been demonstrated to correlate with tumor progression.^71–73^ COX-2 gene knockout significantly decreased adenoma formation in a murine colorectal polyposis model and evidence from population-based longitudinal studies suggests that long-term usage of aspirin, a COX-2 inhibitor, is correlated with a decreased incidence of colorectal cancer.^74,75^ In an early immunohistochemistry study of resected sporadic and NF2-related VS by Hong et al.,^76^ COX-2 was expressed in almost all tumors and across tumors its expression level correlated with the Ki67 cellular proliferation index. More recently Behling et al.^77^ undertook a large microarray study of tissue samples from 1048 vestibular schwannomas, including 111 NF2-related VS, in order to analyze the expression of COX-2 and its relationship with both the proliferation marker MIB1 and clinical data such as tumor size and prior anti-inflammatory medication usage. While there was no association between COX2 or MIB1 expression and the prior use of either nonsteroidal anti-inflammatory drugs, glucocorticoids, or other immunosuppressant agents, COX-2 expression was associated with increasing tumor size and higher cellular proliferation rates as measured through MIB1 immunostaining.^77^ While the exact cellular origin of the elevated proliferation indices seen within these high COX2-expressing tumors was not identified by the authors, it can be hypothesized, based on the findings of Lewis et al.,^19^ that an increased abundance of proliferating macrophages was the source of this relationship.^77^

COX-2 is known to interact with a number of downstream mediators including the NF-κB pathway and inhibitors of COX-2 such as aspirin have been shown to reduce activation of NF-κB.^78^ Furthermore, celecoxib, a COX-2-specific inhibitor, is able to induce apoptosis in colon cancer cell lines by inhibiting 3-phosphoinositide-dependent kinase 1 activity,^79^ an enzyme not only involved in activation of NF-κB,^80^ but which also phosphorylates and activates AKT a serine/threonine kinase previously shown to promote VS tumor growth.^80,81^ Prostaglandin E2 (PGE2), however, is the end product of COX-2-regulated arachidonic acid metabolism most frequently implicated in carcinogenesis. PGE2 has been demonstrated to have both pro-inflammatory and pro-angiogenic effects and among its many effects it is known to stimulate macrophage production of the pro-inflammatory cytokines IL-1 and IL-6.^82^ PGE2 has also been shown to stimulate the secretion of CCL2, a chemokine implicated in macrophage infiltration and promotion of macrophage differentiation toward an alternatively activated, pro-tumorigenic M2 phenotype.^82,83^ A number of cytokines stimulated by COX-2 such as IL-6 are themselves inducers of COX-2 activity and were considered in the context of previous analyses of cytokine profiles in VS.^34,38,44^ The possibility of a constitutively active, autocrine signaling loop that drives chronic inflammation within VS, leading to macrophage infiltration and macrophage polarization is therefore a possibility.^20,38^ Evidence of such a positive feedback loop within the brain has been identified in the formation of intracranial aneurysms, whereby the inflammation induced by the aberrant COX-2 expression was found to be mediated by NF-κB.^84^

## In Vivo Biomarkers of Inflammation in VS

Given the growing evidence that the immune response plays a key role in VS growth, a key concern is how this intratumoral inflammation can be detected and quantified in vivo. Identification of such a biomarker would be of critical importance in any future clinical trial, having the potential to not only permit earlier detection of VS growth but also allow specific targeting of immunomodulatory therapy to tumors with high intratumoral inflammation. Previous authors have investigated the neutrophil-to-lymphocyte blood ratio (NLR) as a potential marker of subclinical inflammation in patients with extracranial tumors and found that a higher NLR is associated with adverse overall survival.^85,86^ In a study of 161 patients with sporadic VS, Kontorinis et al.^87^ measured the peripheral blood NLR ratio within 12 months of initial diagnosis and found that compared to static tumors NLR values were higher in the growing VS group and that the NLR ratio was a good independent predictor of tumor growth status. While the authors argued that the findings of this study supported the theory of subclinical inflammation as an underlying mechanism for VS growth,^87^ their study was limited by its retrospective nature and the lack of assessment of changes in NLR ratio values over time. Future prospective studies should be undertaken to assess whether peripheral blood markers of inflammation such as the NLR ratio or plasma cytokine expression profiles can be used as biomarkers of intratumoral inflammation and tumor growth.

**Fig. 4:**
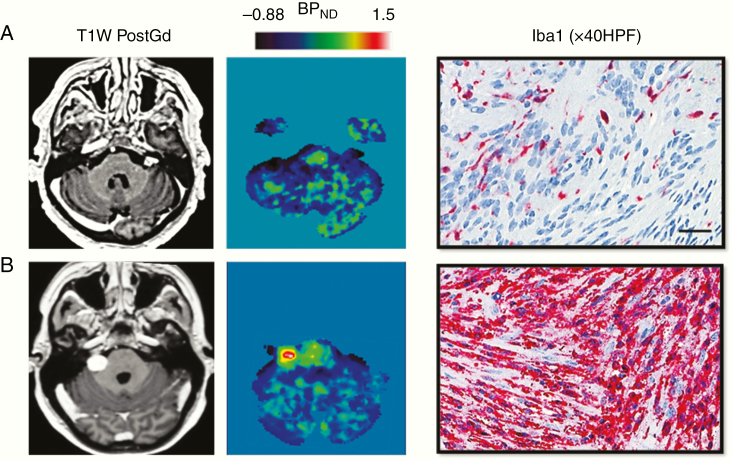
[^11^C]-(*R*)-PK11195 PET as a biomarker of intratumoral inflammation in vestibular schwannoma (VS). Representative imaging and histology from a patient with a static left-sided VS (A) and a growing right-sided VS (B) are shown. Specific binding of the TSPO PET tracer, [^11^C]-(*R*)-PK11195, is demonstrably higher within the growing VS compared to the static tumor. Comparative immunohistochemistry (Iba1 red, immunoperoxidase) demonstrates that the source of this increased specific binding within the growing VS was an abundance of intratumoral Iba1+ macrophages.

Recent studies have also sought to establish whether novel imaging biomarkers could be used to detect local intratumoral inflammation in VS. Breun et al.^46^ in a study of 60 NF2-related and sporadic VS demonstrated that the chemokine receptor CXCR4 is upregulated at both the protein and mRNA transcript level within tumoral Schwann cells and have recently demonstrated in a pilot PET/CT of 6 tumors that the CXCR4 PET ligand [^68^Ga]Pentixafor demonstrates uptake in VS.^88^ The largest in vivo imaging study of inflammation in VS to date, however, was undertaken by Lewis et al.^19^ using [^11^C]-(R)PK11195, an established TSPO PET tracer for inflammation.^89^ In this prospective study, the authors demonstrated that compared to static tumors, growing sporadic VS displayed higher specific binding of [^11^C]-(R) PK11195 and that the source of this increased specific binding within growing tumors was an abundance of intratumoral Iba1+ macrophages (Figure 4). Analysis of concomitantly acquired dynamic contrast-enhanced (DCE) MRI data demonstrated that growing tumors also displayed significantly higher mean *K*^trans^, a DCE-MRI derived measure of vascular permeability.^19,90^ Indeed derived *K*^trans^ values correlated strongly with both [^11^C]-(R)PK11195 specific binding and macrophage density suggesting that MR-derived permeability metrics could also serve as future more clinically applicable biomarkers of inflammation in this tumor group. There have been no studies investigating PET imaging biomarkers of inflammation such as [^11^C]-(R)PK11195 within NF2-related VS but a comparative study of DCE-MRI metrics in 21 NF2-related tumors and 24 sporadic tumors demonstrated that both sporadic and NF2-related VS show marked similarities with regard to their DCE-MRI derived microvascular metrics and that similar to sporadic tumors, within NF2-related VS, there was a close association between tumor vascularity and macrophage abundance (Lewis et al., unpublished, 2019).

## Inflammation as a Therapeutic Target

As the evidence for inflammation as a key process in the progression of VS has emerged relatively recently, there are few examples of attempts to specifically target this process in patients with these tumors. Indeed, the paucity of studies targeting inflammation as the driver of growth in VS highlights the emerging nature of this research field. To date, the only targeted molecular therapy for VS that has been successfully translated into clinical use is the angiogenesis targeting antibody bevacizumab (Avastin).^7^ Previous studies have suggested that a possible mechanism for the rapid effect of anti-angiogenic therapy in VS is a reduction in peritumoral blood vessel permeability or “vascular normalization” effect^91–93^ and a recent in vivo MRI study supports this hypothesis, demonstrating reductions in both the vascular permeability metric *K*^trans^ and tumoral free water content following bevacizumab (Avastin) therapy in responding VS.^92^ Based on observations from both in vivo imaging and ex vivo tissue studies of the close association between vascular permeability and cellular inflammation in VS,^18,19^ it can be hypothesized that this vascular normalization also leads to concomitant reductions in intratumoral cellular inflammation.

Some authors have suggested that aspirin usage may prevent VS growth, reporting in some small retrospective case series lower VS growth rates among aspirin users after controlling for age and gender.^94,95^ Larger, more recent case series have, however, challenged this view, finding no evidence that aspirin usage is associated with reduced VS progression.^96,97^ The reason for these contradictory results is not clear but their retrospective and correlative nature makes them vulnerable to discrepancies in both aspirin dosing and patient self-reported usage across studies.^94,96^ The Congress of Neurological Surgeons have nonetheless recently recommended the administration of aspirin for patients with vestibular schwannomas as an antiproliferative treatment^98^ and a recently commenced randomized phase II clinical trial of aspirin in VS will hopefully help better clarify the drug’s position as a preventive therapy for VS growth.^99^

The demonstration that there is upregulation of candidate cytokines such as IL-1, IL-6, and TNF-α in VS tissue is an important one as many safe, effective, and well-tolerated immunomodulatory agents are already in current clinical usage against these mediators. Larger studies to validate these findings are required, but this raises the possibility that well-established immune-targeting agents already in clinical use could be re-purposed against VS and fast-tracked through to early phase II and III studies. There is nonetheless a danger that by focusing on just a few candidate cytokines and chemokines, other potentially more important targetable molecular pathways in these tumors may be ignored. It is of equal importance therefore, that alongside this a “bottom-up” approach is also adopted in future studies, whereby the key molecular pathways driving the inflammatory milieu at both the genetic and proteomic level in VS are characterized in detail.

## Conclusion and Future Directions

In this review, we have provided an overview of the contribution of inflammation to the pathophysiology of VS and shown that there is growing evidence from both ex vivo and in vivo studies that inflammation is a key feature of the microenvironment in these tumors. Indeed recent research has shown that far from representing a localized proliferation of Schwann cells these tumors should instead be viewed as a complex immune microenvironment characterized by macrophage infiltration, inflammatory cytokine expression, and constitutive activation of varied pro-inflammatory molecular pathways.^18,38,44,60,69^ Our understanding of this inflammatory microenvironment is currently at an early stage and a number of key questions remain such as how primary genetic alterations within the neoplastic Schwann cell population translate to the formation of this inflammatory milieu in vivo. Nonetheless, with current advances in single-cell genomics and advanced proteomic analysis techniques, it is hoped that future studies will address these questions and characterize the key inflammatory pathways within VS at both the cellular and subcellular level. The advent of novel orthotopic animal models of VS tumorigenesis^100^ will also hopefully permit not only a greater understanding of how the immune microenvironment arises and evolves over time in these tumors but also serve as a valuable in vivo substrate for testing candidate immunomodulatory agents. Through such approaches and the establishment of candidate biomarkers of inflammation in these tumors, it is hoped that novel immune-related therapeutic targets can be carried forward to early-phase clinical trials and ultimately adopted as a new therapeutic weapon against these challenging and enigmatic tumors.

## Funding

This work was supported by funding from the Cancer Research UK, (C8742/A18097) and the Dowager Countess Eleanor Peel Trust.

*Conflict of interest statement.* No conflicts of interest to declare.

Authorship Statement. Conception: O.P. Manuscript drafting: C.H., D.L., and C.O’L. Manuscript review: C.H., D.L., C.O’L., C.A.D., D.G.E., F.R., D.B., A.T.K., D.C., and O.P.
